# Experimental Study of Straw-Based Eco-Panel Using a Small Ignition Initiator

**DOI:** 10.3390/polym13081344

**Published:** 2021-04-20

**Authors:** Linda Makovicka Osvaldova, Iveta Markova, Stanislav Jochim, Jan Bares

**Affiliations:** 1 Department of Fire Engineering, Faculty of Security Engineering, University of Zilina, Univerzitna 1, 010 26 Zilina, Slovakia; iveta.markova@fbi.uniza.sk; 2 Department of Wooden Constructions, Faculty of Wood Science and Technology, Technical University in Zvolen, T.G. Masaryka 24, 960 01 Zvolen, Slovakia; jochim@tuzvo.sk; 3 EKOPANELY SERVIS s.r.o., Jedousov 64, 535 01 Prelouc, Czech Republic; bares@ekopanely.cz

**Keywords:** eco-panel, small ignition initiator, straw, relative burning rate, weight loss, fire properties

## Abstract

Straw, a natural cellulose-based material, has become part of building elements. Eco-panels, compressed straw in a cardboard casing, is used as building insulation. Eco-panel is a secondary product with excellent insulating properties. If suitably fire-treated (insulation and covering), straw panels’ fire resistance may be increased. This contribution deals with monitoring the behavior of eco-panels exposed to a small ignition initiator (flame). The samples consisted of compressed straw boards coated with a 40 mm thick cardboard. Samples were exposed to a flame for 5 and 10 min. The influence of the selected factors (size of the board, orientation of flame with the sample) were compared on the basis of experimentally obtained data: mass loss. The results obtained do not show a statistically significant influence of the position of the sample and the initiating source (flame). The results presented in the article confirm the justifiability of fire tests. As the results of the experiments prove, the position of a small burner for igniting such material is also important. Such weakness of the material can also be eliminated by design solutions in the construction. The experiment on larger samples also confirmed the justifiability of fire tests along with the need for flame retardancy of such material for its safe application in construction.

## 1. Introduction

Eco-panel represents a natural material used in the construction industry [1,2]. Eco-panel is a fully recyclable material and, as a building element, is a secondary product made out of straw with a casing of recycled paperboard or cardboard [3]. Eco-panel is produced by pressing straw at 180–220 °C [4], using pressure (15 MPa), without any binders (pure straw core), and subsequently gluing it together on both sides with recycled cardboard or recycled paper [5,6]. A disadvantage of this material is that it is relatively heavy [7,8]. 

Eco-panel has excellent thermal insulation properties and low thermal conductivity [9,10]. The research on eco-panels has focused primarily on monitoring the quality of indoor climates in enclosed environments with such straw insulation [11], and a solution of low-energy wood houses insulated with eco-panel. Sadzevicius [12] determined, by standard methods, the main qualities of straw: heat conductivity, fire, and humidity resistance, durability, and strength of compressed straw. The compressive strength of straw panels, calculated by averaging, yielded a value of 144 ± 30 kPa [13]. They assert that there is no standard for compressive strength of panels, including straw [13].

The growing need for sustainable products and the stringent legislative requirements related to the hazardous formaldehyde emissions from wood-based panels have boosted scientific and industrial interest in the production of eco-friendly, wood-based panels and optimal utilization of the available lignocellulosic materials [14,15].

The fire characteristics of building materials play an important role in building design. Fire properties of natural materials are usually, in comparison with “standard” materials, worse [16].

The importance of correctly applying natural materials, such as straw, in constructions is illustrated by the fire in the construction of the Alternative Theater S2, built under an overpass in Zilina. The construction consisted of wooden bottle crates, straw bales (the inner lining made of a thousand straw bales), and clay [17]. The fire, which originated at 1:30 a.m. on 19 May 2019, left the structure completely destroyed. The fire spread quickly and the flames also damaged the construction of the overpass.

### Fire Characteristics of Straw-Based Eco-Panel Constructions Coated with Cardboard

Fire characteristics of straw structures are a topic of discussion. Straw structures are said to be very susceptible to fire and burn very well [16,18]. 

There are separate technologies for straw burning as a form of renewable energy production (including in the form of whole bales) [19,20]. Straw has a higher specific calorific value than brown coal (4.9 kWh.kg^−1^ of dry matter or 4.0 kWh.kg^−1^ of straw with a moisture content of 15%) [21]. It is used as a heating element in many countries, not only for environmental reasons, but also because of its low cost [22,23]. The burning of straw is influenced by its chemical nature—a natural organic polymer. Xie et al. [24] carried out a comparative analysis of thermal oxidative decomposition and fire characteristics for different straw powders using thermogravimetry and cone calorimetry. The fire characteristics of straw are very similar to those of wood. The thermal degradation of straw starts at 270 °C, occurs in two steps, and the final temperature is 600 °C [24]. 

**Figure 1:**
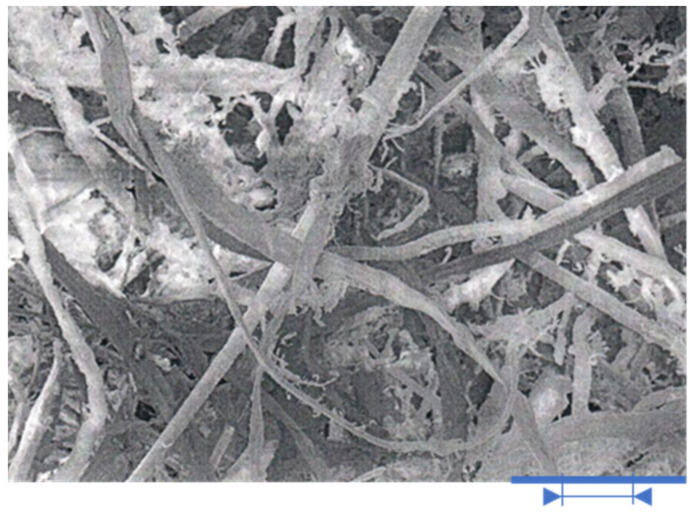
Light microscopy images of straw fibers with magnification 200×. Legend: Blue line presents a size of 100 microns (μm) in 2D layout.

Straw breaks down (Figure 1) and in this case increases the area of the flammable substance that can enter the oxygen reaction, which is significant because the presence of an oxidizer is crucial to continued burning. 

Eco-panels are formed from pressed straw enclosed in a surface material; the pressed, tightly packed nature of the straw prevents the oxidative agent from reacting with the thermally degraded layer.

The results of several tests and measurements of straw structures intended to define its fire characteristics show that straw constructions with a proper coating have excellent values of fire resistance [16,25,26,27,28,29]. Surface treatments on straw walls (e.g., clay plaster, plasterboard cladding, and lime plaster) can increase the fire resistance of structural components [29,30]. Field and laboratory tests carried out by Theis [31] show plastered bale walls to be highly resistant to fire damage, flame spread, and combustion [31].

Cardboard treatment represents a decisive factor. Producers of eco-panels assert that there is a self-extinguishing effect [10,16] because of the highly pressed straw in the eco panel core, which contains a minimum amount of air. If an eco-panel burns, the paper burns first (Cardboard) followed by the straw, which slows down or even stops the process [32].

The improvement of the fire performance of straw and other similar materials can be achieved in two ways—by physical and chemical retardation. Physical retardation is based on regulating the size of the input material and the output density of the material. Chemical retardation is possible by applying a retardant to the input material, thus making a “new” product out of it, or by adding a retardant during the manufacturing process. All of the above-mentioned procedures can be carried out for eco panels.

The purpose of this article is to monitor the fire behavior and define the fire properties of eco-panels, defined as a pressed straw core with cardboard surface treatment used in wooden constructions, when exposed to flame. The article also deals with non-fire-retarded material in order to obtain input parameters for potential further observations.

## 2. Materials and Methods

### 2.1. Test Samples

**Figure 2:**
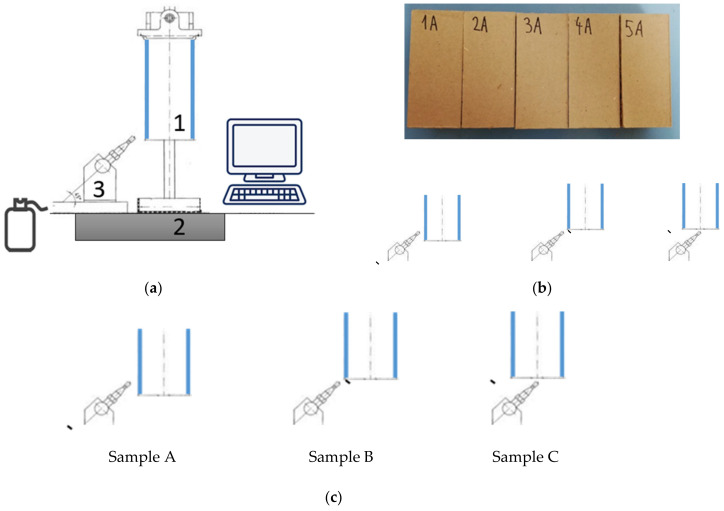
(**a**) Test equipment scheme; (**b**) First set of samples (A); (**c**) position of the burner relative to sample A, to sample B, to sample C. Legend for Figure 2. (**a**): 1—test sample, blue lines represent cardboard, 2—scales monitoring mass loss, 3—flame burner, in position of sample A.

Two groups of samples, 30 mm thick, were used in the experiment (Table 1). The first set of samples were 50 × 100 mm in size and included 3 groups of 5 samples, marked A, B, and C (Figure 2c). The second group, marked D, were samples 100 × 200 mm in size (Figure 4). All samples were conditioned for 24 h in a burning laboratory and their weight was determined. As the sample size increased, the weight differences became more significant (Table 1). 

The experiment was carried out in laboratory conditions with an ambient temperature of 21.5 °C, air flow 0.02 m·s^−1^, and ambient humidity 57%.

### 2.2. The Ignitability Fire Test by a Small-Time Attack Flame of Eco Panel

The experiments were carried out in the fire-chemical laboratory of the Faculty of Security Engineering at the University of Zilina in Zilina. Samples A, B, and C were tested on a non-certified device (Figure 2a). The ignitability fire test by a small-time attack flame of construction products was carried out in the special fire box. The test sample was mounted in a vertical position.

Samples A, B, and C were exposed to a small ignition initiator, a flame, for 5 min. The distance between the flame and the surface of the sample was maintained at 15 mm during experiments. The position of the burner, however, changed for each set of samples (see Table 1 and Figure 2b).

**Figure 3:**
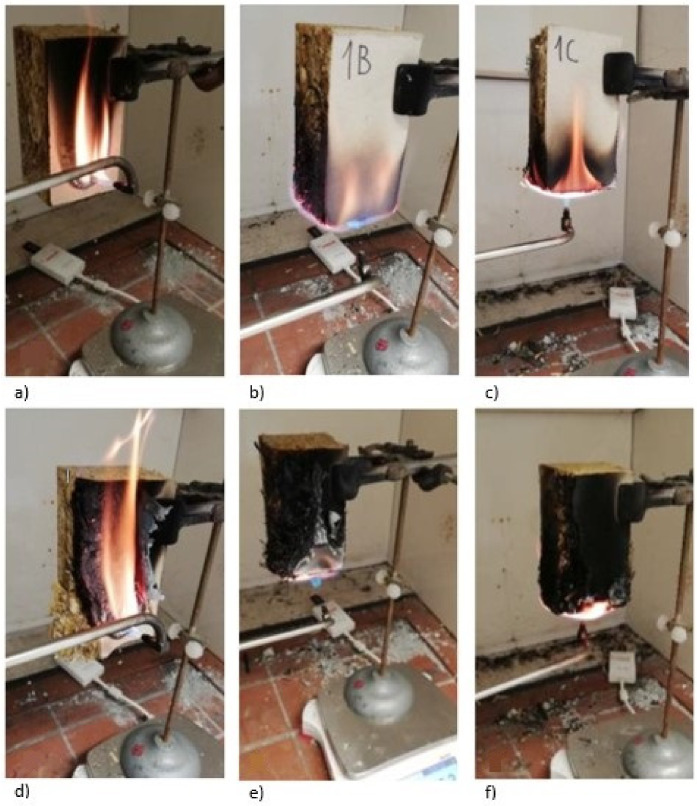
Location of the burner in the experiments in the first minute (**a**–**c**) and 3rd minute (**d**–**f**) for samples A (**a**,**d**); B (**b**,**e**); C (**c**,**f**).

The samples of group A were exposed to a flame perpendicular to the lateral side of the eco panel, with the flame touching the surface of the cardboard of the eco-panel carton, approximately 1 cm from the bottom edge (illustration Figure 3a,d).

Group B samples were exposed to a flame on the edge of the eco panel, such that the flame acted on both the cardboard and pressed straw (example Figure 3b,e).

Group C samples were exposed to flame from below, directly onto the pressed straw (demonstration Figure 3c,f).

### 2.3. Test Evaluation

The test evaluated flame spread along the surface of the sample to 100 mm (A, B, and C) or 200 mm (D) vertically from the point of contact with the flame. The results indicate whether the flame spread over the sample (Yes) or it did not (No). Furthermore, the time for which flame spread occurred was recorded, as was mass loss of the samples.

Two test methods based on different evaluation principles were used to assess the experiment. The first focused on the propagation of the flame over the surface of the material, so the samples were in a vertical position. In this case, the position of the source that ignited the material was important. The fire source was the flame of a gas burner (pure propane) with a flame length of 20 mm. As previously mentioned, in order to achieve a comprehensive evaluation of the material, testing of its ignition in several positions of the flame and the sample was carried out (see Table 1 and Figure 2). Position A represents the effect of the flame on the surface of the sample (40 mm) from its lower edge (Figure 3a,d). Position B represents the action of the flame on the edge of the material, so that the flame acted on both the paperboard and the pressed straw (example Figure 3b,e). In Position C, the flame acted on the center of the sample within its width and thickness. Group C samples were exposed to the flame from below directly on the pressed straw (demonstration, Figure 3c,f). These burner positions revealed the weaknesses in the material. Samples A, B, and C were exposed to a small ignition initiator, a flame, for 5 min. During the experiments, the distance between the flame and the sample surface was maintained at 15 mm.

**Figure 4:**
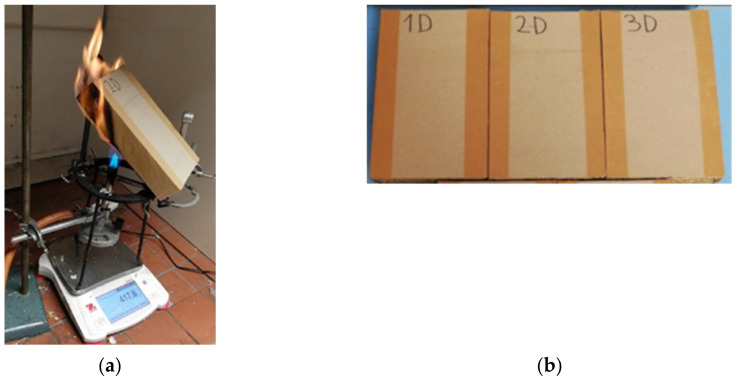
(**a**) Treatment of the experiment for group D samples. (**b**) Samples D.

The second experiment monitored the actual burning (flame propagation) using the D samples (200 × 100 × 10 mm). The higher intensity flame from the Bunsen burner acted on the center of the sample along its length and width, and the sample was placed at an angle of 45° to the horizontal plane (see Figure 4). The flame loading time of the sample was 10 min. The flame size was 100 mm and the position of the burner orifice from the center of the sample was 90 mm. Evaluation criteria of individual experiments are stated in Section 2.4.

### 2.4. Weight Loss and Relative Burning Rate

When the samples were exposed to heat, we observed and recorded weight loss in 10 s intervals. Relative weight loss was calculated according to the relation (1) [33]:

(1) $$\delta_{m}\left(\tau \right)=\frac{\Delta m}{m\left(\tau \right)}\cdot 100=\frac{m\left(\tau \right)-m\left(\tau +\Delta \tau \right)}{m\left(\tau \right)}\cdot 100\left(\%\right)$$

where Δ*_m_*(*τ*) is relative weight loss in time (*τ*) (%), *m*(*τ*) is sample weight in time (*τ*) (*g*), *m*(*τ* + Δ*τ*) is sample weight in time (*τ* + Δ*τ*) (g), Δ*m* is weight difference (*g*).

Relative burning rate has been determined according to the following relations (2) [33] and (3) [33]:

(2) $$v_{r}=\left|\frac{\partial \delta_{m}}{\delta \tau }\right|\left(\%/s\right)$$

or numerically

(3) $$v_{r}=\frac{\left|\delta_{m}\left(\tau \right)-\delta_{m}\left(\tau +\Delta \tau \right)\right|}{\Delta \tau }\left(\%/s\right)$$

where *v_r_* is relative burning rate (%/s), Δ*_m_*(*τ*) is relative weight loss in time (*τ*) (%), Δ*_m_*(*τ* + Δ*τ*) is relative weight loss in time (*τ* + Δ*τ*) (%), Δ*τ* is time interval where the weights are subtracted (s).

### 2.5. Mathematical and Statistical Processing and Evaluation of Results

To evaluate the influence of burner position on the thermal degradation of the samples using mass loss, the results obtained were subjected to statistical analysis. The results regarding mass loss were statistically evaluated by a single-factor variation analysis (ANOVA) using the LSD test (confidence intervals of 95%, 99%, using STATGRAPHICS version 18/19 software), where the factor of influence was the contact point flame vs. sample based on the different positions of the burner.

## 3. Results

**Figure 5:**
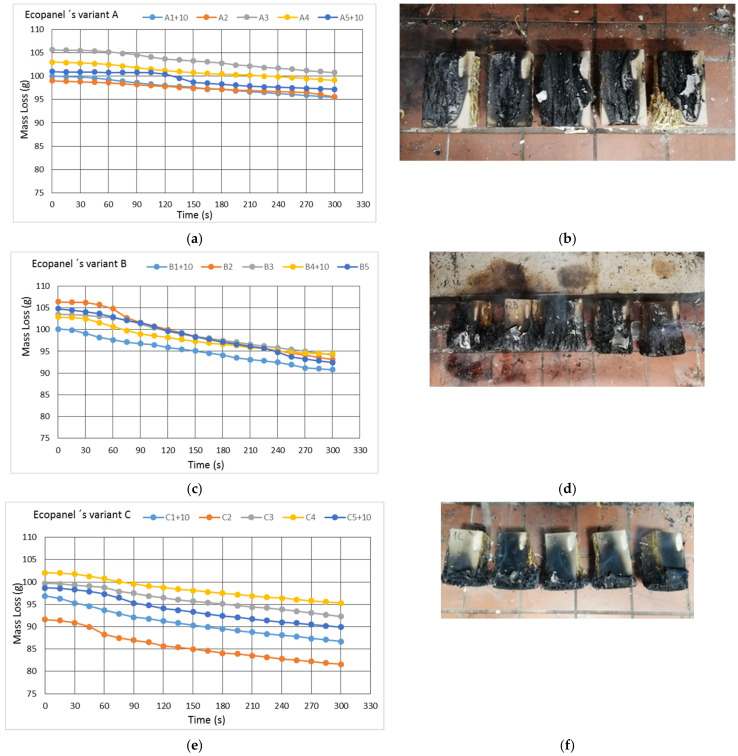
Graphical dependences of mass loss during experiment of (**a**) Group A samples, (**c**) Group B samples, (**e**) Group C samples. Samples after the experiment (**b**) Group A samples, (**d**) Group B sample, (**f**) Group D samples. Exposure time was 5 min.

The experimental data obtained using mass loss in relation to exposure time (Figure 5) were used to calculate the burning rate.

**Figure 6:**
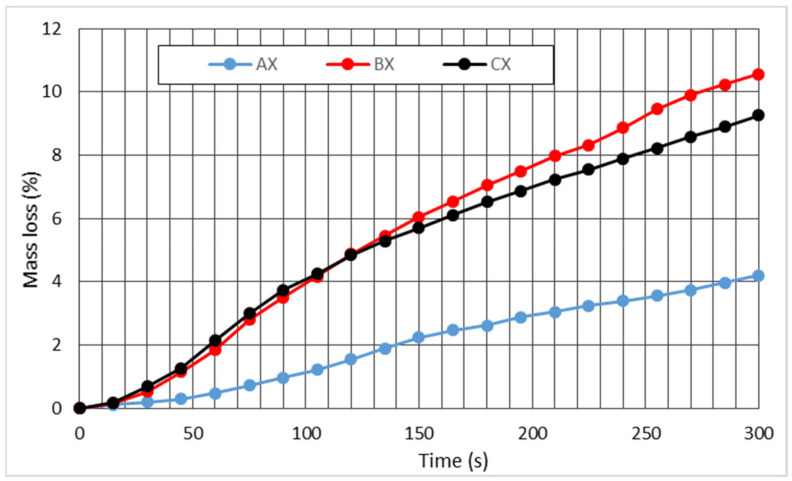
Mutual comparison of the increase in mass loss of eco panels in groups A, B, and C based on time of burning. Note: The value X represents the average value of all samples in each group.

All variants exposed to flame ignited. The variability of the results depend on the straw sample and its preparation method (such as applying glue on cardboard, etc.). The flame position and the point of contact between the flame and the sample had an effect on the results. The mass loss courses differed (Figure 5a,c,e). This influenced the change in mass loss, most notably for the sample B (Figure 6), in which the flame was located on the edge of the panel, so both the straw and the cardboard were exposed to flame.

Samples of group C burned in a relatively similar way; direct exposure of the straw to flame caused the slowest burning progress and the lowest rates of fire propagation (Figure 5e). During the 5-min test, the samples burned halfway (Figure 5f) and retained their shape.

When the eco panel burns, a lower mass loss occurs compared to massive wood [34]. Maximum mass loss for sample B is 10.05% (Figure 6).

**Figure 7:**
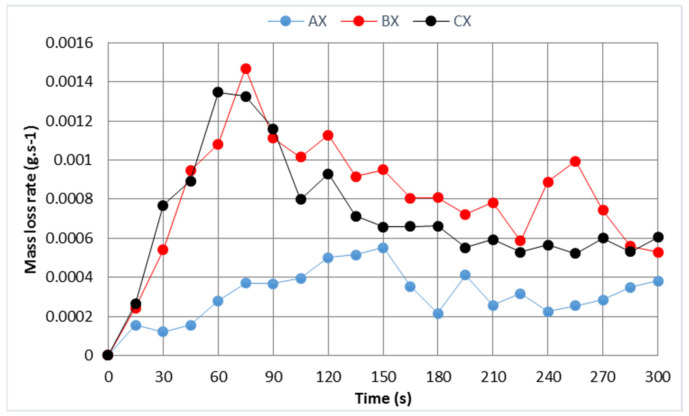
This is a figure. Schemes follow the same formatting. Note: The value X represents the average value of all samples in each group.

The burning rate is determined as the mass loss over time. The time-dependent course of the burning rate is confirmed by the most intense burning behavior of sample B. The lowest burning rate was observed for the A samples (Figure 7). The assumption that group C samples would the lowest mass values was not confirmed. At the same time, the burning rate for these straw eco panels was one-tenth lower than the burning rate of wood. The Kacikova and Makovicka [35] survey, which monitored the burning rate of juniper, spruce, fir, and larch exposed to a heat initiator, resulted in values in the range of 0.038–0.080 (mg·s^−1^). At the same time, the curves show a similar course to those showing the heat release rate (HRR) over time [24,36]. The eco-panel samples’ burning rate curve have a regular course, and the rate of the burning rate in the initial phase does not have a sharp increase. The effect is probably slowed down due to the smooth surface of the cardboard. Subsequently, the case burned through and the straw started to burn (150 s), and there was a drop in the burning rate due to a loss of homogeneity of the combustible material.

**Figure 8:**
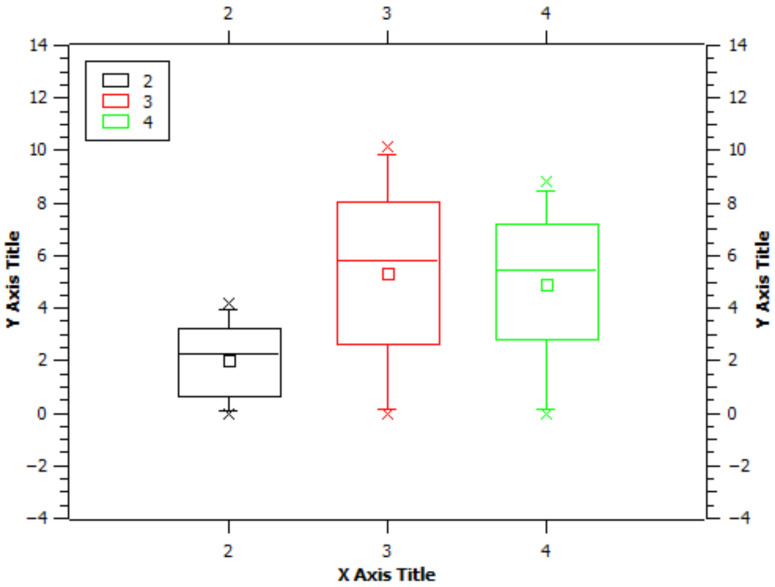
Graphical representation of the statistical evaluation of the influence of the burner’s position on the mass loss of the sample when eco-panels in groups A, B, and C were exposed to the flame. Legend: X Axis Title = Samples. (2-Group A samples; 3-Group B samples; 4-Group C samples.) Y Axis Title = Mass Loss.

The application of a single-factor ANOVA (STATGRAPHICS version 18/19 software) analysis did not confirm a significant difference in ignition time based on panel thickness. (Table 2; Figure 8). 

### Statistical Results: The Null Hypothesis Applies

**Figure 9:**
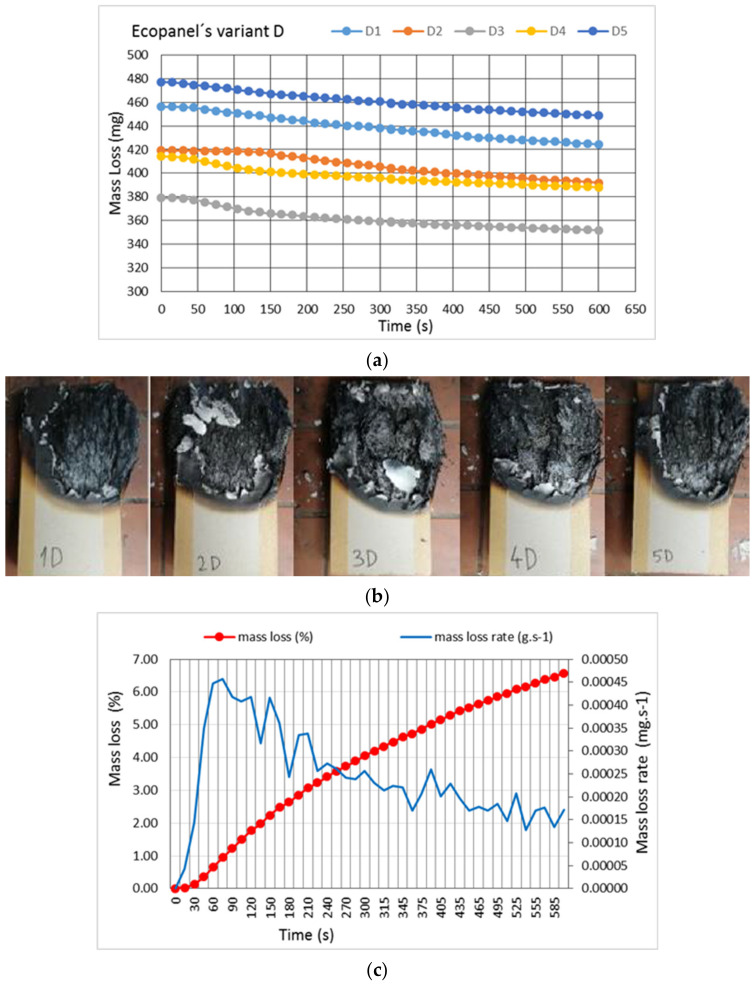
(**a**) Weight loss of D samples over time; (**b**) Group D samples after the experiment, exposure time 10 min; (**c**) The increase in mass loss and the burning rate of D samples. All charts display average values of all the samples.

Another aim of the research was to follow the behavior of samples of larger dimensions and simulate the position of roofing sleepers in wooden houses. The samples were placed at an angle of 45° and the burner was placed into the center of the sample. The burning time was 10 min, the standard time of fire-fighting units’ arrival. The results of the burning behavior of samples in group D show reduced mass loss and maintain the original sample dimensions (Figure 9). During the experiment, the flame operated on the surface and did not reach the inner part of the panel. In all samples, the cardboard layer burned through and straw began to burn. The process was slower in comparison to the A, B, and C samples, and the maximum value of weight loss was 6.4% (Figure 9c). At the same time, the burning rate decreased by one-tenth compared to A, B, and C samples.

Several authors [11,16,37] conducted research into eco-panels as construction elements according to the Fire Reaction Test methodology. They used the ignitability fire test by a small time attack flame and concluded that crushed straw might not be classified into class E according to its reaction to fire [16]. Class E is a category of products which are capable of resisting a small flame without substantial flame spread, for a short time [38,39]. The Class D category, however, is defined as products which satisfy the criteria for class E and are also capable of resisting a small flame without substantial flame spread for a longer period. In addition, they are also capable of undergoing thermal attack by a single burning item with sufficiently delayed and limited heat release [39]. Whether crushed straw could be classified into Class D must be confirmed by another series of fire tests.

Eco-panel, a straw building element, is characterized by a potential generation of dangerous gases. This is caused by imperfect burning of straw [40,41]. Jenkins [42] presents the research of pollutant emission factors for open field burning of wood and rice straw as comparable. They differ only in the production of CO; in wood it is 5.54%, of dry fuel, and in rice straw 3.22% of dry fuel.

## 4. Conclusions

The tests were performed on eco panel board samples with small dimensions and the results correspond to their dimensions. Eco panel (straw with cardboard casing) thermally degrades and burns with a luminous flame. As a result of exposure to the flame, initiation, and burning occur at rates slower than the standard values of the burning of wood (as a structure). A side effect is the instability of the eco panel. The samples gradually disintegrate, and parts of the straw fly off. One of the priority conclusions is the insufficient size of test specimens A, B, and C. For an objective assessment of the specimens, minimum dimensions of the tested sample 180 × 90 × 40 mm as specified in EN 13501-2 + A1 [43] are necessary. As sample D was twice as big as the other samples, it had a significantly different, slowed, burning (slowed down) course over a doubled time of exposure. In tests with large dimensions of eco panel boards (2.8 × 3 m) in wooden building structures, they showed a fire resistance of 120 min, which is declared sufficient by the Fire resistance test report [43].

The presentation of one part of the research results, which deals with monitoring the fire characteristics of natural materials applicable in building structures, shows the risk of thermal decomposition of eco panels, but above all, shows the importance of size, shape, and positioning of the eco panel in the structure in order to increase fire resistance.

## Figures and Tables

**Table 1:** Test samples.

Series	Group	Composition	Dimensions (mm)	Weight (g)	Burner Position	Test Time (min)
1.	A	Straw Cardboard	50 × 100	101.74 ± 2.381	Lateral side of panel	5
	B			103.74 ± 1.785	Edge of panel	5
	C			99.74 ± 1.998	Bottom of panel	5
2.	D		100 × 200	429.14 ± 34.180	Below 45°	10

**Table 2:** One-way ANOVA for statistical evaluation of the influence of the burner’s position on the burning rate (weight loss) of the sample when Eco-panels in groups A, B, and C were exposed to a flame.

**Sample**	**Number **	**Mean**	**Standard Deviation**	**Variance**	**Standard Error**
Time	100	157.7	86.9299	7559.8181	8.6929
A	100	2.1363	1.4263	2.0343	0.1426
B	100	0.0008	0.0004	1.8864	4.3432
C	100	5.1528	2.9357	8.6183	0.2935
**Source**	**Df**	**Sum of Squares**	**Mean Square**	**F Value**	***p* Value**
Model (Between groups)	3	1,804,842.948	601,614.3160	318.0002	4.6151
Error	369	749,179.6158	1,891.8677		
Total	399	2,554,022.5639			

Null Hypothesis: Means of all selected data sets are equal. Alternative Hypothesis: Means of one or more selected datasets are different. At the 0.05 level, the population means are significantly different.

## Data Availability

Not applicable.
